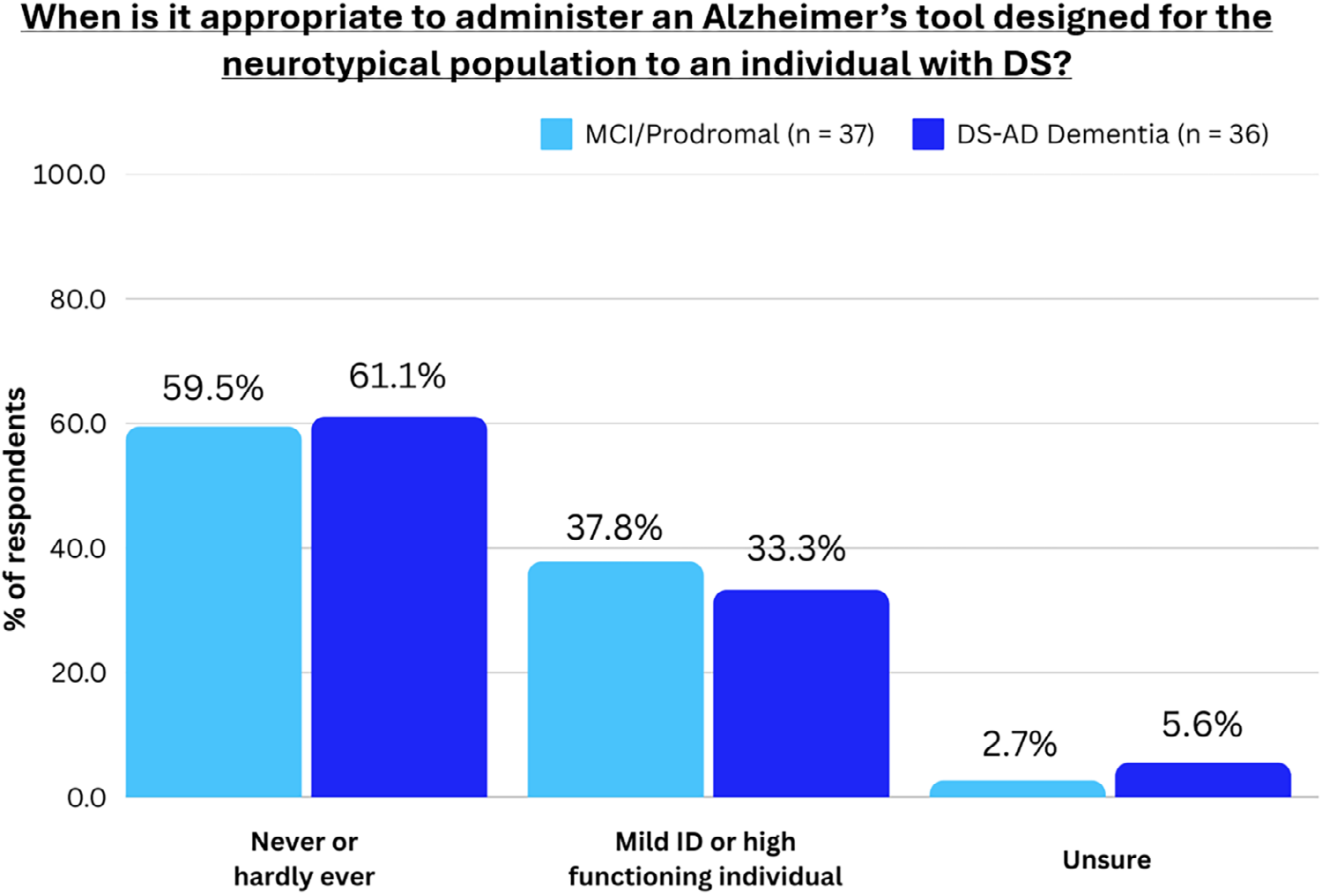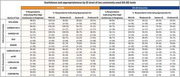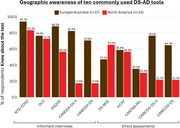# Assessment Tools for DS‐AD: Global Expert Perspectives on Utility and Appropriateness

**DOI:** 10.1002/alz70857_106815

**Published:** 2025-12-25

**Authors:** Amit Das, Matthew P. Janicki, Natalia S. Rozas, Stephanie L. Santoro, Hampus Hillerstrom

**Affiliations:** ^1^ LuMind IDSC Foundation, Woburn, MA, USA; ^2^ National Task Group / ID & Dementia Practices, Rockport, ME, USA; ^3^ University of Illinois at Chicago, Rockport, Maine, ME, USA; ^4^ Massachusetts General Research Institute, Boston, MA, USA

## Abstract

**Background:**

People with Down syndrome (DS) have a significantly higher risk for Alzheimer's disease (AD) than the neurotypical (NT) population. Due to intellectual disability (ID) inherent in DS, detection of cognitive decline in DS‐associated AD (DS‐AD) requires specialized tools. This study examined international perspectives on the utility and application of these tools.

**Method:**

A survey was conducted with 51 international DS‐AD clinicians and researchers, of which 43 complete submissions were analyzed. The survey queried topics related to clinical staging of MCI‐DS and DS‐AD Dementia, including questions about the utility and appropriateness of ten commonly‐used DS‐AD assessment tools (five informant‐based, five direct assessments).

**Result:**

Over 95% of respondents stated that for detecting both MCI‐DS and DS‐AD Dementia, tools designed for the NT population should never be used or only be considered for individuals with mild ID. In the detection of MCI‐DS/DS‐AD Dementia, informant interviews were viewed as moderately‐to‐highly useful on average by 82.2%/93.9% of respondents, appropriate for mild ID by 99.2%/97.2%, appropriate for moderate ID by 95.1%/92.4%, appropriate for severe ID by 63.6%/71.2%, and appropriate for profound ID by 34.5%/35.6%. In the detection of MCI‐DS/DS‐AD Dementia, direct assessments were viewed as moderately‐to‐highly useful on average by 85.1%/91.7% of respondents, appropriate for mild ID by 96.3%/96.8%, appropriate for moderate ID by 88.5%/90.8%, appropriate for severe ID by 17.4%/24.6%, and appropriate for profound ID by 4.1%/5.9%. Six tools were generally more known by EU and Australian respondents, while one was generally more known by North American respondents.

**Conclusion:**

Clear consensus emerged that the use of AD tools designed for the NT population has little or limited utility when diagnosing DS‐AD, especially for individuals with moderate‐to‐severe ID, emphasizing the need for specialized DS tools. Informant‐based assessments were more readily recommended across all severity levels of ID, whereas direct assessments were deemed suitable primarily for mild‐to‐moderate ID. Regional differences in tool awareness suggest that broader global consensus regarding use and equivalency of established instruments is needed. Therefore, bridging these knowledge gaps through an internationally agreed‐upon framework clarifying which specialized DS tools to use—and for what purposes—remains critical for consistent, evidence‐based assessment and care.